# Evolving Therapy for Celiac Disease

**DOI:** 10.3389/fped.2019.00193

**Published:** 2019-05-14

**Authors:** Shakira Yoosuf, Govind K. Makharia

**Affiliations:** Department of Gastroenterology and Human Nutrition, All India Institute of Medical Sciences, New Delhi, India

**Keywords:** prolyl endopeptidase (PEP), glucocorticoids, exocrine pancreatic insufficiency, immune tolerogenesis, gluten, genetically modified wheat, helminth therapy, larazotide acetate

## Abstract

Gluten is known to be the main triggering factor for celiac disease (CeD), an immune-mediated disorder. CeD is therefore managed using a strict and lifelong gluten-free diet (GFD), the only effective treatment available currently. However, the GFD is restrictive. Hence, efforts are being made to explore alternative therapies. Based on their mechanisms of action on various molecular targets involved in the pathogenesis of CeD, these therapies may be classified into one of the following five broad approaches. The first approach focuses on decreasing the immunogenic content of gluten, using strategies like genetically modified wheat, intra-intestinal gluten digestion using glutenases, microwave thermal treatment of hydrated wheat kernels, and gluten pretreatment with either bacterial/ fungal derived endopeptidases or microbial transglutaminase. The second approach involves sequestering gluten in the gut lumen before it is digested into immunogenic peptides and absorbed, using binder drugs like polymer p(HEMA-co-SS), single chain fragment variable (scFv), and anti- gluten antibody AGY. The third approach aims to prevent uptake of digested gluten through intestinal epithelial tight junctions, using a zonulin antagonist. The fourth approach involves tissue transglutaminase (tTG) inhibitors to prevent the enhancement of immunogenicity of digested gluten by the intestinal tTG enzyme. The fifth approach seeks to prevent downstream immune activation after uptake of gluten immunogenic peptides through the intestinal mucosal epithelial layer. Examples include HLA-DQ2 blockers that prevent presentation of gluten derived- antigens by dendritic cells to T cells, immune- tolerizing therapies like the vaccine Nexvax2 and TIMP-Glia, cathepsin inhibitors, immunosuppressants like corticosteroids, azathioprine etc., and anti-cytokine agents targeting TNF-α and interleukin-15. Apart from these approaches, research is being done to evaluate the effectiveness of probiotics/prebiotics, helminth therapy using *Necator americanus*, low FODMAP diet, and pancreatic enzyme supplementation in CeD symptom control; however, the mechanisms by which they play a beneficial role in CeD are yet to be clearly established. Overall, although many therapies being explored are still in the pre-clinical phase, some like the zonulin antagonist, immune tolerizing therapies and glutenases have reached phase II/III clinical trials. While these potential options appear exciting, currently they may at best be used to supplement rather than supplant the GFD.

## Introduction

Celiac disease (CeD) affects 0.7% of the global population (1). Although initially believed to affect only the intestine, it is now considered to be a systemic autoimmune disease. The consequent spectrum of manifestations of CeD is wide, ranging from gastrointestinal and nutritional derangements resulting from the enteropathy to neuropsychiatric symptoms, infertility and liver diseases, among extra-intestinal manifestations (2). For the affected individuals (3) and their care providers (4, 5), it can adversely affect the quality of life.

Since the discovery of dietary gluten as the causative agent six decades ago (6), our comprehension of the pathophysiology of CeD has grown substantially. This process has been facilitated by documented clinical observations as well as exponential advancements in immunology and molecular medicine. However, the cornerstone of management of CeD is based on the lesson learnt from that discovery itself, and it still remains the gluten- free diet (GFD). Patients may find the GFD and related lifestyle modification to be burdensome, due to its poor satisfactoriness, unavailability and higher costs (7). In fact, hyper- vigilance to GFD adherence can also affect quality of life (8), much like the disease itself. This has in turn created an unmet need for alternatives (9). Also, there may be patients who remain persistently symptomatic despite a GFD, in whom adjunct therapies may be required (10). Herein, we have reviewed the principles of potential, alternative dietary and non-dietary therapeutic strategies and their current status of investigation (Supplementary Table 1).

## Pathogenesis

In a broad sense, the pathogenesis of CeD may be considered to be a result of the interplay of genetic susceptibility and immunological factors, described as follows.

### Genetic Basis

CeD is caused by autoimmunity to gluten in genetically predisposed individuals, with frequently observed familial clustering (11). The main implicated alleles are the HLA-DQ2 and HLA-DQ8, that contribute to 30–50% of genetic susceptibility (12). Genotyping studies have also identified non-HLA variants associated with CeD, with most genes in these variants involved in the structure or function of immune cells (13–16). However, the non-HLA genes carry only a modest increase in the risk of CeD (17). Regardless, all these genes represent potential targets for therapeutics. Some of them, along with their respective loci are summarized in Table 1.

**Table 1:** Common gene loci involved in celiac disease.

**Locus**	**Gene**	**Chromosome**	**References**
CELIAC1	HLA-DQ2 and HLA-DQ8	6p21.3	(15)
CELIAC2		5q31-33	(14)
CELIAC3	Intergenic between CD28, CTLA4 and ICOS	2q33	(16)
CELIAC4	MYO9XB (myosin IXB gene)	19p13.1	(13)

### Immunogenicity of Gluten

**Figure 1:**
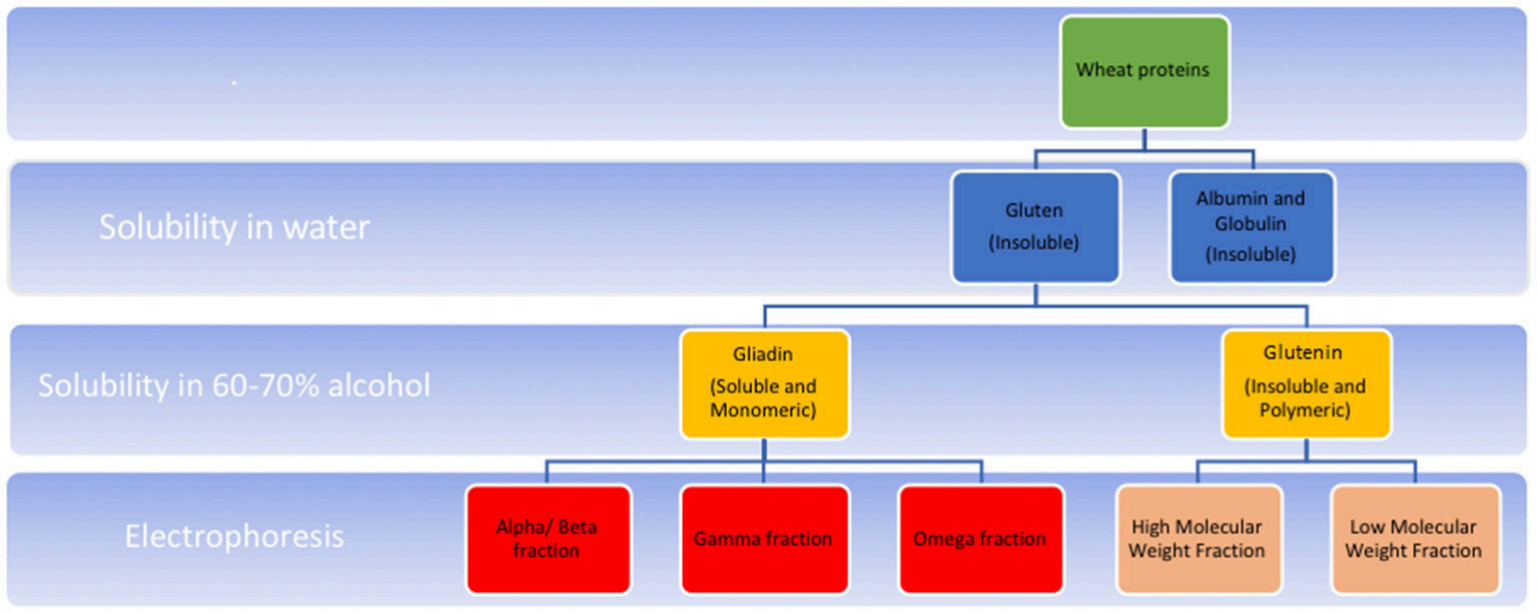
Schematic representation of composition of wheat gluten. Gluten refers to the water insoluble protein component left after washing wheat flour. In various cereals, gluten is a conglomerate of peptides, composed of two main fractions-prolamins (known as gliadins in wheat), and glutelins (known as glutenins in wheat). These fractions differ in their solubility in alcohol; the former of these is alcohol soluble. Also, prolamins occur in monomeric form with intrachain disulphide bridges formed by cysteine residues. Glutelins, in contrast, occur as polymers formed by interchain disulphide bridges in addition to intrachain bridges. Gliadin is composed of further subfractions-α/β, γ (each having intrachain disulphide bridges) and ω (having no disulphide bridges) which differ in their electrophoretic mobilities in a low pH medium. Glutenin fraction, upon reduction of the interchain bridges, in turn yield high molecular weight (HMW), and low molecular weight (LMW) fractions depending on their mobility on SDS PAGE electrophoresis. High molecular weight fractions of glutelin have less disulphide bonds compared to the low molecular weight fractions.

Gluten is found in wheat as well as barley, rye and oats. Gluten is composed of two peptide conglomerates viz. glutelins and prolamins (18). Of these, prolamins are the gluten components that are implicated in CeD; they are found variously in different grains as gliadins in wheat, secalins in rye, a mix of both in triticale, hordeins in barley, and avenins in oats. In wheat, gliadins are in turn composed of sub-fractions–α/β, γ, ω1, ω2, and ω5 (Figure 1). The α-gliadin sub-fraction has the maximum immunogenicity in CeD, contributing the most to toxic epitopes upon digestion (19).

At the molecular level, gliadins are made predominantly of multiple glutamine (35%) amino acid residues linked to proline (15%) (20). Presence of proline makes the structure of prolamins complex as well as sterically inaccessible/ resistant to proteolytic enzymes of the human stomach and intestine. Consequently, these luminal proteases only succeed in digesting the prolamins into larger oligopeptides. These oligopeptides are immunogenic in CeD (21) and some of them include, the most immunotoxic, 33- mer peptide 57–89 (with the amino acid sequence LQLQPFPQPQLPYPQPLPYPQPQLPYPQPQPF), and relatively less immunogenic ones like peptide 57–73, peptide 111–130, 26- mer peptide with the sequence FLQPQQPFPQQPQQPYPQQPQQPFPQ), peptide 31-43 etc. (22–27).

**Figure 2:**
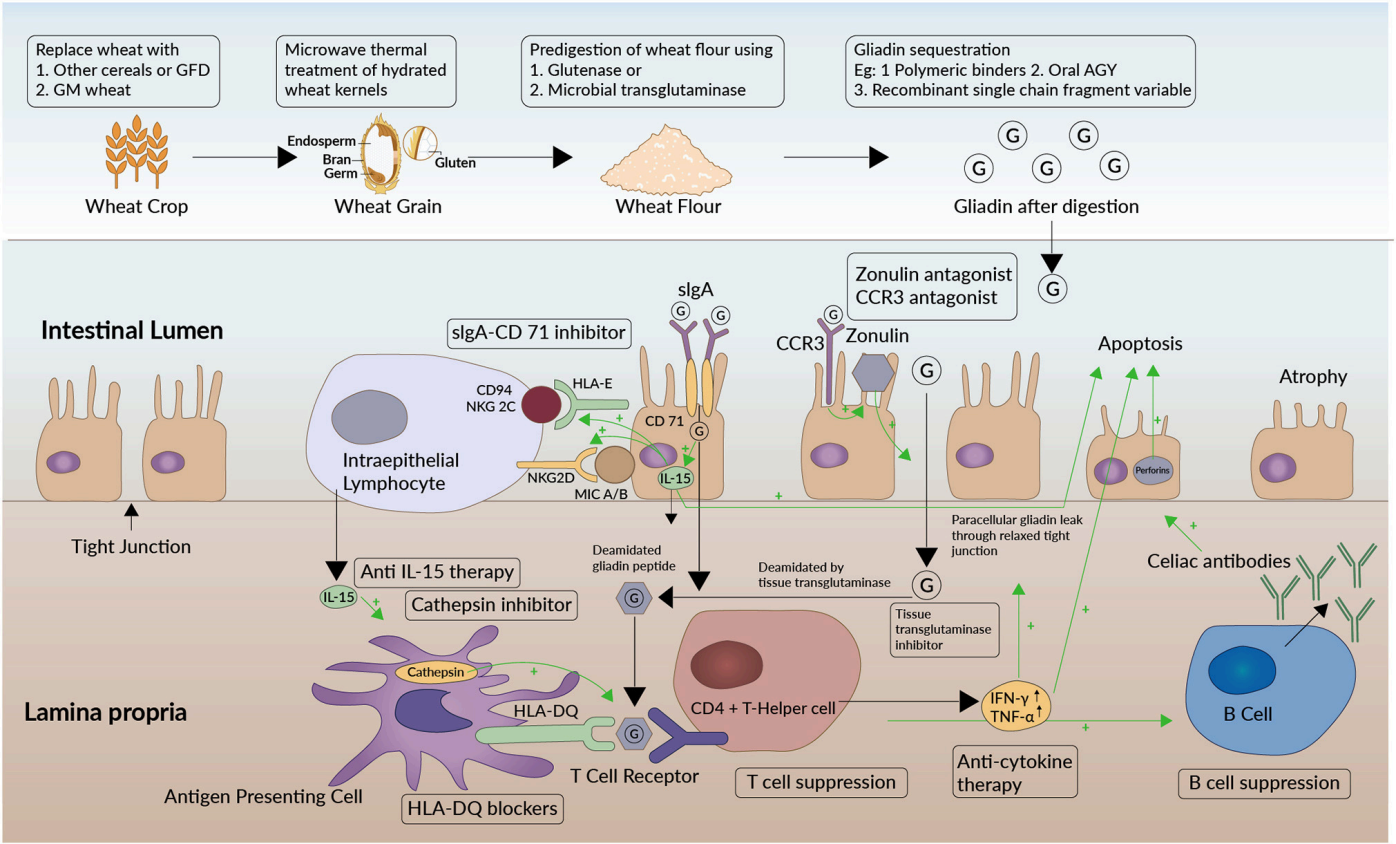
Target sites of therapeutics along the pathogenetic pathway of celiac disease. Sites of action of therapeutic approaches under investigation (enclosed in black boxes) are shown at different levels of the pathogenetic pathway of celiac disease. Green arrows in the figure depict a stimulatory effect. The oligomers (G) formed from gluten digestion enter the lamina propria of the small intestine across the epithelial barrier. They do so by a paracellular pathway that involves the protein zonulin. Zonulin is structurally similar to the zona occludens toxin expressed by *Vibrio cholera* and regulates epithelial permeability at apical tight junctions. In the zonulin pathway, gluten products attach to the chemokine receptor CXCR3 on the luminal aspect of the intestinal epithelium that increases the formation of zonulin. The zonulin then relaxes the interepithelial tight junctions through the PAR2/EGFR (Protease activated receptor 2/Epithelial Growth Factor Receptor) pathway. This increased permeability in turn leads to influx of gliadin. An alternative pathway implicated in gliadin uptake is the transcellular pathway involving secretory IgA (Immunoglobluin A) and CD71. CD71 or transferrin receptor is found to be expressed in higher amounts on the luminal aspect of intestinal epithelial cells in CeD. The CD71 co- localizes with secretory IgA and has been postulated to promote transcellular gliadin uptake into the lamina propria in CeD. Zonulin antagonists, CXCR3 antagonists, and sIgA/CD71 pathway antagonists would prevent gliadin transport through either of these two pathways. Once the gluten immunogenic epitopes are transferred into the lamina propria, the HLA-DQ2 and -DQ8 bearing antigen presenting cells (APCs) recognize the epitopes. Consequently, APCs activate CD4+ helper T cells, setting off an inflammatory cascade. Cathepsins play a role in processing the antigens in APCs and promoting the interaction between APCs and CD4+ T cells. Cytokines like IFN-γ and TNF-α are released by activated CD4+ cells, which further aggravate this permeability and facilitate a self-propagating mechanism. T-cells also activate B-cells which mature to produce antibodies against gluten and tissue transglutaminase−2 (celiac antibodies). HLA-DQ blockers, anti-cytokine therapy, and cathepsin inhibitors are some of the therapeutic approaches being explored. In addition, the tissue transglutaminase-2 (tTG-2) enzyme deamidates the glutamine residues to glutamate in gliadin. The resulting deamidated gliadin peptides are more immunogenic. Also, by virtue of the relatively large size of these partially digested, negatively charged proline containing fragments, they tend to settle and form bonds with neighboring tissues resulting in immobilized neoepitopes. Thus, these peptides form immunoreactive autoantigens and enteropathy ensues. tTg-2 enzyme inhibitors are being explored as therapeutic options. The immunotoxicity is mediated by the increased production of interleukin-15 (IL-15) by the intestinal epithelial cells as well as by the intraepithelial lymphocytes (IEL) in CeD. IL-15 upregulates the receptor NKG2D (Natural Killer Group) expression on IEL which interacts with MIC-A and MIC-B (MHC class I polypeptide-related sequence A and B) displayed on epithelial cells. IELs in patients with CeD also express an NK receptor called CD94/NKG2C. CD94/NKG2C recognizes HLA-E, a protein that is upregulated in epithelial cells in response to Interferon- γ (IFN-γ). The interaction of these two ligand- receptor pairs activates the IELs and triggers them to kill epithelial cells through perforins. Among other therapeutic options, gluten from wheat may be rendered non-immunogenic by genetic modification. Attenuation of immunotoxicity has also been attempted using microwave energy application on hydrated wheat kernels or by gluten modification either using glutenases from fungi or bacteria or using microbial transglutaminase. Alternatively, gliadin in gluten may be sequestered in the intestinal lumen using polymeric binders, AGY or oral Immunoglobulin Y, and recombinant single chain Fragment variable.

The digested gliadin peptides enter the lamina propria of the small intestine across the epithelial barrier, by a paracellular pathway that involves the protein zonulin (Figure 2). Zonulin is structurally similar to the zona occludens toxin associated with *Vibrio cholera* and has been observed to be a controller of epithelial permeability. In the zonulin pathway, gliadin products attach to the chemokine receptor CXCR3 on the luminal aspect of the intestinal epithelium. CXCR3 in turn increases the formation of zonulin, which relaxes the inter-epithelial tight junctions through the PAR2/EGFR (Protease activated receptor 2/Epithelial Growth Factor Receptor) pathway. This increased permeability leads to influx of gliadin (28). An alternative pathway implicated in gliadin uptake is the transcellular pathway. This involves secretory Immunoglobulin A (IgA) that co-localizes with another molecule, the CD71 to promote transcellular uptake of gliadin products into the lamina propria (29). CD71 is the transferrin receptor, but is found to be expressed in higher amounts on the luminal aspect of intestinal epithelial cells in CeD.

The tissue transglutaminase-2 (tTG-2) enzyme modifies the digested gluten immunogenic peptides that have entered the mucosa, by deamidating their glutamine residues to glutamate. These negatively charged glutamate side chains have a higher potential to be recognized as immunogenic. Also, by virtue of the relatively large size of these partially digested proline containing fragments, and the negative charge of glutamate, they tend to settle and form bonds with the neighboring extracellular matrix, resulting in immobilized neoepitopes. The formation of these bonds may be directly catalyzed by the tTG (30). Ultimately, all the gluten- derived antigens are recognized and processed by the HLA-DQ2 and -DQ8 bearing antigen presenting cells (APCs), which activate CD4+ helper T cells, setting off an inflammatory cascade. Activated CD4+ cells release cytokines like Interferon- γ (IFN-γ) and Tumor Necrosis Factor-α (TNF-α), thereby further enhancing the permeability and facilitating a self-propagating mechanism of gliadin uptake. T-cells also activate B-cells which mature to produce antibodies against gluten and tissue transglutaminase-2 (celiac antibodies). These antibodies further contribute to the ensuing immune- mediated enteropathy. As well, the immunotoxicity is mediated through the increased production of interleukin-15 (IL-15) by the intestinal epithelial cells and the intraepithelial lymphocytes (IEL). IL-15 upregulates the receptor Natural Killer Group 2D (NKG2D) expression on IEL which interacts with MHC class I polypeptide-related sequence A and B (MIC-A and MIC-B) ligands displayed on epithelial cells (31). IELs also express an NK receptor called CD94/NKG2C. CD94/NKG2C recognizes Human Leukocyte Antigen-E (HLA-E), a protein that is upregulated in epithelial cells in response to IFN-γ in CeD (32). The interaction of these two ligand-receptor pairs activates the IELs and triggers them to kill epithelial cells through perforins, and other mechanisms (33).

## Novel Therapies for Celiac Disease

Our understanding of the pathogenetic pathway of CeD has provided the context for the development of new drugs that target different aspects of this pathway. An overview of the mechanisms of action of these drugs is given in Figure 2. The therapeutic options for CeD (Supplementary Table 1) currently being investigated can be broadly classified as belonging to one of the following approaches.

### Approach 1: Decreasing Immunogenic Epitopes in the Gluten

#### Genetically Modified Wheat

Bread wheat (*Triticum aestivum*) has a hexaploid genome AABBDD, wherein chromosomes 1 and 6 predominantly harbor the genes known to code for immunotoxic components of gluten (34). Attempts have been made to manipulate these genes to attenuate immunotoxicity. However, doing so may also alter the gastronomic properties of wheat, the yield etc, if these properties are governed by the same or neighboring genetic loci. Although studies have explored the genetic manipulation of these chromosomes, a variant that is both safe in CeD and suitable for commercial production is yet to be found.

A study explored a variant formed by the removal of genes on chromosome 1 that code for β, γ, and ω gliadin fractions. While the toxicity was attenuated, the mechanical properties of wheat were not altered. However, when α fraction was attenuated instead, the mechanical properties were compromised while also significantly reducing the dose of immunogenic T cell epitopes (35).

Prolamins from a wheat variant called C173 were tested *in vitro* on intestinal epithelial cells derived from CeD patients. This variant was formed by the removal of predominantly toxic epitopes in gliadin fractions viz. Gli-A2, Gli-D1, and Gli-D3. There was no worsening of villous: crypt (V:C) ratio, however there was an increase in pro- inflammatory cytokines like IFN-γ, TNF-α as well as in anti- tTG antibody levels in the collected supernatant (36). Therefore, this was unlikely to be suitable for further clinical testing in CeD.

In another recent development, the International Wheat Genome Sequencing Consortium delivered a high-quality annotated reference genome sequence of the Chinese spring wheat (37). The sequence is referred to as the RefSeq v1.0. The process started in 2005 and has now yielded the sequence of 107,891 high-confidence genes, including the genomic context of their regulatory sequences. This has the potential to fast track development of genetically engineered wheat with attenuated immunotoxicity while preserving its gastronomic or agronomic properties (38).

#### Intraluminal Digestion of Gluten Using Oral Glutenases

In order for gliadin-derived oligomers to enter the lamina propria and still not induce an immune reaction, the oligomers should contain nine amino acids or less (39). Therefore, gluten degrading enzymes (glutenases) that digest gliadin into peptides with nine or less amino acid moieties have been explored as a therapeutic option in CeD. Most of these are glutamine and proline specific enzymes, since these are the principal amino acids found in immunogenic motifs of gluten. The forthcoming sections will highlight some of these enzymes.

The glutenase EP-B2 (endoprotease B, isoform 2) is a glutamine specific peptidase. Being a cysteine protease enzyme, it has a Cys-His-Asn catalytic triad in its active site. It is secreted naturally in the acidic endosperm of germinating barley seeds (*Hordeum vulgare*) where it serves to digest hordein, the analog of gliadin. Glutenase EP-B2 is optimally active at low pH, resistant to pepsin but lysed at physiological concentrations of trypsin, and has good specificity for the sequence QXP, which is abundant in the 33-mer as well as other immunotoxic gluten sequences. These factors make it a good fit for therapy in CeD as a gastric active enzyme (40). Gass et al. studied its action in animal models. They observed the complete digestion and liquefaction of a gluten containing meal into a viscous fluid in gliadin-sensitized Wistar rats that were fed this enzyme along with the meal. The stomachs of the rats that did not get the enzyme contained a relatively solid, dry paste-like material post digestion of the same meal. This effect was even more evident at higher doses and extended digestion times (41). One of the concerns with any gastric-based glutenase is that if the gastric emptying occurs before the complete digestion of gluten, immunotoxic residue would reach the duodenum and induce the disease. It is therefore reasonable to combine this enzyme with other glutenases which are stable in the gastric environment but start acting when the food chyme reaches duodenum, preempting any immunotoxicity in the intestinal epithelium.

Proline specific endoproteases (PEP) from the microbes *Flavobacterium meningosepticum* (FM-PEP)*, Sphingomonas capsulata* (SC-PEP), and *Myxococcus xanthus* (MX-PEP) have also been investigated as potential glutenases. They are serine proteases and each has a larger β-propeller domain and a smaller, N-terminal catalytic domain that breaks the peptide bond of proline residues at the carboxy end of the gluten protein (42). Activity of SC-PEP extends into the acidic range of pH, and is by and large, unaltered in the presence of pepsin (43). However, the other two PEPs are lysed by pepsin. Furthermore, FM-PEP is inactivated by the small intestinal enzyme trypsin in the presence of bile acids. MX-PEP too is unstable in the presence of bile salts (44). Considering these limitations, SC-PEP has been explored as a more favorable candidate for therapy in CeD. In order to improve its action further, mutant variants (variant 10,224 or 10,230) of SC-PEP have been developed which have 200-fold higher resistance to pepsin and 20% higher turnover at acidic pH (45).

While SC-PEP has high specificity for gluten immunogenic epitopes, it has relatively low specificity for long peptide sequences. This is because the larger β-propeller domain preferentially allows smaller gliadin fragments into the active site, and therefore, is unable to completely eliminate the immunogenic gliadin peptides. This limitation could be overcome by combining it with other enzymes with complementary specificity. Combination of EP-B2 with SC-PEP, for instance has been explored for application in CeD. The EP-B2 efficiently digests the 33 mer peptides into smaller, not necessarily non-toxic proline containing fragments. The PEP complements its action by digesting the proline- glutamine links in these smaller oligopeptides, thereby reducing their immunotoxicity. Gass tested this combination on rat models and found that 1:1 ratio of this oral enzyme combination was more efficacious than either enzyme alone in reducing immunotoxic oligopeptides from gliadin (46).

Several clinical trials have been conducted to assess the effectiveness of this enzyme mixture in making dietary gluten safe for patients with CeD. One of the most prominent of these has been using the enzyme cocktail ALV 003, now known as latiglutenase. Latiglutenase is a proprietary, 1:1 combination of EP-B2, or ALV 001 plus PEP, or ALV 002. A randomized control trial (RCT) studied CeD patients on GFD, who received a diet containing gluten (16 g/day for 3 days) pre-treated with either ALV003 or placebo (NCT00859391). The ALV003 group showed significantly lower immunological activation, as seen on peripheral T cell IFN-γ responses to gliadin (47). A phase 2a RCT (NCT00959114) on 41 CeD patients on GFD, found that on gluten challenge (with 2 g bread crumb for 6 weeks), V:C ratio deteriorated significantly less in patients treated with ALV003 than with placebo. However, intraepithelial CD3^+^ lymphocytes remained unchanged in the ALV003-treated patients compared to placebo-treated patients. This study was also significant for having used bread crumbs to simulate real life gluten intake as opposed to pre-digested gluten (48). Although the aforementioned clinical trials were promising, the results of the most recent large-scale phase 2 b trial (NCT01917630) were disappointing. Four hundred ninety CeD patients who were symptomatic despite GFD for a year, were included in a dose ranging, placebo-controlled double-blinded study. In a modified intention-to-treat analysis, no significant differences were observed on histological, serological or symptomatic end-points (49). In a *post-hoc* analysis of data, it was found that there was significant reduction in the abdominal symptoms in those patients who were seropositive for celiac antibody. The authors concluded that seronegative patients did not experience any symptomatic improvement, possibly because their symptoms may be attributable to non-celiac causes (50). Another phase 2 study is underway to test the effect of ALV003 on histopathological parameters of 80 CeD patients who had been adherent to a GFD (NCT03585478). Overall, therefore, the effectiveness of ALV003 remains to be established.

Kuma030 is an engineered glutenase developed by the Institute for Protein Design, University of Washington. Prior to its development, the investigators identified a naturally occurring enzyme from the acidophilic microbe Alicyclobacillus sendaiensis, called kumamolisin-As (KumaWT). It is a serine endoprotease, with optimal activity over the pH range of 2–4/37°C and therefore adaptable for use in the gastric environment (51). Based on the structure of KumaWT, an enzyme was designed, with specificity toward known gliadin peptides. The new enzyme called KumaMax or Kuma010, had 116 times higher proteolytic activity, and 877 times higher specificity for the target gliadin oligopeptides. This enzyme was further modified to result in several-fold higher activity against immunogenic 33-mer and 26-mer peptides. This version was called Kuma030. In comparison to SC PEP- EP B2, Kuma030 seemed to be more efficient. At the highest concentration of the PEP-EP B2 i.e. 1:10 weight/weight (w/w) ratio, gluten was degraded by 84.4%. Kuma030 at a lesser concentration of 1:40 w/w ratio achieved >99.9% gliadin degradation thereby reducing the gluten content to 3 ppm, well-below the 20 ppm threshold for “gluten-free” labeling. When gluten sensitive T cells isolated from patients with CeD were incubated with gliadin pre-treated with Kuma030, a dose dependent reduction in IFN-γ production and T cell proliferation was observed (52). In light of these findings Kuma030 may hold promise in the near future.

AN-PEP (*Aspergillus niger-* Prolyl Endopeptidase), like SC-PEP, is a glutenase with optimum activity at gastric pH between 3 and 5. It is also similarly resistant to proteolysis by pepsin, but has 60 times faster action compared to another prolyl endopeptidase such as MX-PEP (53). *A.niger*, the fungus from which this enzyme is derived is a food grade microbe, available on an industrial scale. AN-PEP has therefore been explored for suitability as an oral supplement. Being a relatively non-specific enzyme, it has less specificity for immunogenic epitopes on gluten. However, it is highly efficient in degrading gliadin into smaller peptides, and may potentiate action of more specific enzymes if used in combination. It is currently in Phase 2 clinical trials (54). In preliminary studies, the enzyme has been found to catalyze almost complete degradation of gluten epitopes even in complex food matrices such as in fast food meal. Also, co-administration of AN-PEP with gluten to CeD patients led to a complete elimination of T-cell stimulatory peptides from both gliadins and glutenins within 2 h, as measured in their gastric aspirates (55, 56). Another trial (NCT00810654) randomized CeD patients to receive 7 g of gluten along with either placebo or AN-PEP for 14 days. No significant difference in serology, symptoms, or histopathology was observed between the groups, with no patient in either group showing any significant deterioration. However, mucosal IgA tTG deposits were observed in four patients in the placebo group compared to 1 in the AN-PEP group, showing some mitigation of serological response in the latter (57). Trials with bigger samples and longer follow up are warranted for conclusive results on the therapeutic value of the enzyme. Tolerase G is a commercially available form of this enzyme.

Dipeptidyl peptidase- IV (DPP-IV) is an exopeptidase that acts on the amino-terminal side to liberate X-Pro dipeptides in gliadin. It occurs naturally in small amounts in the small intestinal brush border. It has been obtained commercially from the fungus *Aspergillus oryzae* and its potential as a glutenase has been investigated. On its own, DPP-IV has modest efficiency as it can only act on peptides starting with X-Pro. Additionally, DPP-IV has a neutral pH optimum and hence it starts action only in the intestine. Addition of AN-PEP to DPP-IV may however improve its efficiency. The combination when administered as an oral mixture has been found to successfully degrade small amounts of gluten (54). Because of non-specificity of AN-PEP and the very limited proteolytic effect of DPP-IV, the effect of this combination appears to be modest, at best. This combination was studied as a part of STAN1, a cocktail of microbial enzymes commonly used in food supplements. A RCT was done on 35 CeD patients that were persistently seropositive despite GFD. They were randomized to receive either STAN1 or placebo, along with 1 g of gluten per day for a total of 12 weeks. This study found no difference in serology between the two arms (58).

Triticain α, from the papain family, is a cysteine protease and therefore optimally active in the acidic pH. This enzyme possesses collagenase and glutenase activity and occurs naturally as a zymogenic precursor in *Triticum aestivum* (bread wheat). In a gastric like milieu, it exhibits relative resistance to pepsin cleavage, as well as optimal activity at a pH of 3.0/37°C. Upon incubation with gluten, it causes the cleavage of α, γ, ω, and glutenin fractions. However, in presence of trypsin/pH 8.0/37°C, the enzyme is susceptible to destruction. Hence it may be effective only in the gastric digestion of wheat gluten prior to the food bolus reaching the intestine (59). Further *in vitro* testing to study its efficiency as detoxifying enzyme in celiac disease is required. Caricain, another cysteine protease/ prolyl endopeptidase preparation (EC 3.4.22.30) is a derivative of papaya. In a RCT, upon co-administration of caricain with 1 g gluten daily to 20 CeD patients in remission, patients had no worsening of symptom scores and histopathology (60). It is available as a supplement called Gluteguard. Again, more studies would be required to determine its utility in CeD.

Also in nascent stages of investigation are other potential glutenases like the enzyme Pseudolysin (IasB) from *Pseudomonas aeruginosa* isolated from human fecal microbiota (61), the enzyme Nepenthesin from pitcher plants (62) and an unknown enzyme from human salivary plaques (63). In addition to these, there has been a surge of commercially available dietary supplements that claim to aid the digestion of gluten and they are marketed to patients with CeD or to those who prefer to restrict dietary gluten for other reasons. These products are mainly glutenases like DPP-IV, which is known to have limited proteolytic activity. Some of their potential adverse effects, apart from worsening of CeD, include allergy to other components used like egg. None of these products are currently FDA approved (64).

Although all the enzymes described in the preceding sections have been proven glutenase activity under appropriate thermochemical conditions, whether these enzymes completely eliminate all immunogenic epitopes and prevent any possible immune-activation by dietary gluten is the most pertinent question. Moreover, most of the glutenases that are now in advanced stages of clinical trials, have been studied in the context of small amounts of gluten challenge, in patients who are already on the GFD. Such small challenges simulate inadvertent gluten exposure in patients that adhere to GFD, and are useful to study patients that are not responsive despite adherence to GFD. However, the patients who should ideally benefit from these medications are those that find it difficult to adhere to strict GFD or would like to consume near normal levels of gluten in their diet. Whether oral glutenases would help them remains to be proven. Clinical trials with higher doses of gluten challenge are required.

#### Enzymatically Modified Wheat Gluten

##### Gluten modification using glutenases.

Wheat flour can be modified by fermentation with bacteria or fungi. These organisms release proteolytic enzymes that digest gluten, rendering it less toxic, although with modified texture of the flour. This flour can then be mixed with other flours like buckwheat, millets, amaranth etc. to restore its viscoelastic properties.

In a study, sourdough lactobacilli were used to ferment wheat. These organisms release proteases that digest the highly immunogenic 33 mer peptide from gliadin. The species used were *L. alimentarius, L. brevis, L. sanfranciscensis*, and *L. hilgardii*. They were selected previously on the basis of the hydrolytic activities of their enzymes iminopeptidase, dipeptidyl-peptidase prolyl endopeptidase, prolidase, prolinase, and aminopeptidase that are known to digest gluten (65). These bacilli were added to sourdough flour made with wheat (30%), non-toxic oats, millet, and buckwheat and fermented for a day. The impact of ingestion of bread made from this flour on intestinal permeability was assessed in CeD patients using the lactulose/rhamnose excretion ratio. The intestinal permeability was found to remain unchanged as compared to the baseline (66). In another study, feeding a diet of baked goods (8 g of gluten) made from a similar fermented wheat flour, to CeD patients, did not show any impact on the serological, histological, and immunohistochemical parameters (67). Further studies would be required to prove the safety of fermented wheat flour in CeD patients.

A few studies have tried to address the issue of palatability of fermented wheat by adding other flours. Heredia-Sandoval et al. showed that addition of Amaranth flour to enzymatically pre-digested wheat was found to render it acceptable with good viscoelastic properties and at the same time less immune-reactive. The enzyme used in this study was the glutenase AN- PEP (68).

##### Gluten modification using microbial transglutaminase.

Another enzymatic approach that has been investigated is the use of microbial transglutaminase (mTG), isolated from *Streptoverticillium mobaraensis* (69). It is a food grade enzyme and is already extensively used in the food industry to improve the mechanico-chemical properties of food. Unlike the proteolytic enzymes discussed in the preceding sections, it crosslinks gluten molecules in the presence of an amine donor like lysine or lysine methyl ester. It has the same site specificity as the human tTG, but in contrast, lacks deamidase activity, and is not dependent on calcium (70). The enzymatic action of mTG has been hypothesized to attenuate the gluten immunogenic epitopes. Some *in vitro* studies (71), animal studies and *in vivo* studies in the intestinal explants from patients with CeD (72) have suggested reduction of immunogenicity of gliadin in mTG modified wheat flour. In a phase two clinical trial, seven patients each with CeD in remission were fed 100 g of either enzyme modified wheat rusks or modified wheat alone for 90 days. Less number of patients showed elevation in celiac antibodies (2 vs. 4) and worsening of villous abnormalities (1 vs. 4) in those who were fed modified wheat compared to those fed unmodified wheat (69).

Another set of studies have attempted to investigate if the transamidated gluten peptide end products of this enzyme have any immunotoxicity similar to the products of human tTG. For example, Yong et al. showed that the enzyme increases the deamidated end products by 70% when allowed to act on gluten at 40°C and neutral pH (73). Another study found similar results as well (74). Hence, overall, the safety of microbial transglutaminase in CeD is unclear.

#### Thermally Modified Wheat Gluten-Gluten Friendly Bread

Di Luccia et al. (75) developed a technology to detoxify wheat gluten proteins using microwaves (76). Prior to milling, microwave energy is applied for a few seconds to cleaned, hydrated wheat kernels at 15–18% humidity, to reach a high temperature within a short period of time. The process is repeated over several cycles until a temperature of 80–90°C and moisture of 13–13.5% in the grains is reached. After this, grains are dried over 24 h at room temperature and milled. This process had been proposed to attenuate the immunotoxicity of gluten by 99%, as detected by the R5 monoclonal antibody method, which is a method of detection of gluten immunogenic peptides (77). The bread from this flour was called “gluten friendly or GLUFR.” However, a later study found the immunotoxicity of this flour to be unchanged, when checked by the G12 method (another antibody-based gluten immunogenic detection test), mass spectrometry-based proteomics and *in vitro* assay with T cells of celiac subjects (78). It is possible that microwave therapy causes reconfiguration of the gluten structure that interferes with detection of gluten immunogenic peptides by R5 ELISA method. A clinical trial (NCT03137862) evaluating the safety and efficacy of 3 vs. 6 grams of gluten friendly bread for 12 weeks in CeD patients in remission has also been completed, the results however are yet to be published. The results of another *in vivo* study (NCT03168490) evaluating the effect of GLUFR flour on intestinal microbiome and symptoms of patients remain to be seen.

### Approach 2: Intraluminal Sequestration of Gluten Immunogenic Epitopes

#### Polymeric Binders

Polymeric binders are used in certain diseases to sequester toxic compounds in the gastrointestinal tract (79). They have also found application in CeD, where they act by binding gluten in the gut and preventing their breakdown/ absorption. One example is poly (hydroxyethyl methacrylate-*co*-styrene sulfonate) or P(HEMA-*co*-SS), a non-absorbable, high molecular weight, linear co-polymer of hydroxyethylmethacrylate (HEMA), and sodium 4-styrene sulfonate (SS). The experimental form of P(HEMA-*co*-SS), called polymer BL-7010, was studied by Liang et al. They demonstrated the mechanism of the sequestration of alpha gliadin by this polymer using spectroscopic and light scattering methods (80). This was followed by *in vitro* studies that demonstrated no toxic effect of gliadin on cell permeability in the presence of the polymer (81). Subsequent *in vivo* studies using HLA-HCD4/DQ8 mice revealed selectivity in sequestration of gliadin and hordein as compared to other nutrients, as well as reduction in the villous damage caused by the gluten (82).

In order to test practical applicability of industrial grade preparation of the polymer, McCarville and colleagues studied the effectiveness of two batches of BL-7010 together, original polymer A and the industrial preparation polymer B. The latter is structurally similar to the original polymer described by Pinier et al. (81) but has better yield during scaled up industrial preparation. Additional aims were to determine the binding specificity with gliadin and with nutrients, to evaluate the genetic toxicity *in vitro* and the safety and systemic absorption of unlabeled BL-7,010 as part of a toxicology study in mice. For the effectiveness study they prepared transgenic mice deficient in MHC II, expressing HLA-DQ8. They were initially bred on gluten free diet. They were treated with anti-CD25 antibodies to deplete CD4+CD25+Foxp3 cells, which mediate immune tolerance. Then at age 6 weeks onwards they were sensitized with pepsin trypsin digested gliadin and cholera toxin (CT). Post sensitization, mice were administered gluten plus either polymer A or polymer B. It was observed that the polymers bind avidly to the gliadin, with no interaction with vitamins, pepsin, and pancreatin and minimal interaction with albumin. Moreover, systemic absorption was negligible and repeated toxicity studies showed safety in the mice model. While polymer A was completely effective in abrogating villous damage, polymer B was slightly less effective (83).

Phase 1 human studies were completed in October 2014 (NCT01990885), the results of which are yet to be published. The trial involved 40 celiac adult patients who were well-controlled after 6 months of adherence to GFD, and had negative IgA-EMA and IgA-anti tTG antibodies. Escalating, repeated doses of BL-7010 were administered in addition to gluten in a cross over, placebo controlled RCT. The outcomes planned to be assessed were adverse events and safety parameters, as well as plasma levels of BL-7010.

Polymeric binders may be useful to treat inadvertent or minimal gluten exposure and have a potential role, at the very least, as supportive therapy in addition to GFD. Results of human trials with dose variations will throw more light on the extent to which they may be helpful.

#### Anti-gluten Antibody

Chicken egg yolk may be used to produce antibodies to confer passive immunization. IgY is an antibody harvested from the yolk of eggs laid by hens that have been super immunized against gliadin. Gujral et al. showed that these antibodies can be made to significantly neutralize gliadin fraction, in gut- like *in vitro* conditions. They also demonstrated that these IgY antibodies can be put into a capsule form with 50% mannitol. This encapsulated form is called AGY, and it is resistant to degradation in the acidic pH of the stomach. *In vitro* testing showed that gliadin absorption was decreased from 42.8 to 0.7% with the addition of AGY. Also, it was found to be more effective in the presence of food (84). AGY was subsequently tested in a phase one trial to check safety as well as efficacy in improving symptoms of CeD patients who were persistently symptomatic despite adhering to a GFD. AGY capsules were administered along with meals for 6 weeks to the patients. Ten patients completed the study, and no safety concerns were identified. Most patients had fewer celiac symptoms, improved quality of life, lowered antibodies, and lowered lactulose mannitol ratio when taking AGY as compared to the baseline run-in period (85).

The initial results seem encouraging; although testing on a bigger sample size would be required to confirm its benefits. It has minimal toxicity, when administered orally to humans as it does not get absorbed into the systemic circulation to cause systemic immune activation. However, egg allergy would be a contraindication for its use.

#### Single Chain Fragment Variable

Fab is the antigen binding site of an antibody fragment. This Single chain fragment variable (scFv) is a fusion protein that contains the Fab sites of the variable and light chains of the antibody. scFv has been explored for therapeutic use in cancer immunotherapy. It is also being tested *in vitro* for its effectiveness in neutralizing gliadin, for celiac therapy (86). The initial step of the antibody fragment production requires invoking an immune reaction in chicken with gliadin. The birds then serve as a source of RNA with the sequence for the scFv (87). This scFv sequence is subsequently used to produce recombinant scFV in *E. coli* bacteria in larger amounts. Since two antigen binding regions increase binding affinity, two scFv may be joined with a peptide linker to create a tandem scFv (tscFv). This tandem fragment was tested by Eggenreich et al. who found that scFv had the highest affinity of binding to digested gliadin, followed by wheat, and spelt flour. There was no binding with rice and millet flours, indication specificity of binding (86). In order to test this fragment in human subjects, gastric resistant preparations would need to be produced.

### Approach 3: Prevention of Uptake of Gliadin Epitopes

Tight junctions are apical, intercellular junctions that regulate the passage of molecules via the paracellular transport pathway. In normal conditions, pathogenic bacteria, and dietary antigens are prevented from passing through the tight junctions. Changes in the paracellular permeability have been hypothesized to be an early event in the pathogenesis of CeD (88). Immunostimulatory gluten peptides then pass through the paracellular route in such individuals. Human protein zonulin, has been found to be a regulator of epithelial permeability and is highly expressed in the mucosa and blood of patients with celiac disease. It is similar to the zonula occludens toxin (ZOT) expressed by Vibrio cholerae, which impairs integrity of epithelial tight junctions. Gliadin binds to the chemokine receptor CXCR3 releasing Zonulin which subsequently increases the intestinal permeability via the MyD88 dependent pathway (28).

The transcellular gliadin transport pathway can be exploited for management of CeD. Larazotide acetate, a drug targeting Zonulin has been developed by Alba therapeutics, a biopharmaceutical company in USA. Formerly referred to as AT-1001, it is an octapeptide that antagonizes zonulin. Larazotide was found to cause promotion of assembly of actin and E- cadherin around tight junctions of Madin-Darby canine kidney (MDCK) type II cells, thus promoting cell junction integrity (89). Its effect was studied *in vivo* in transgenic HLA HCD4/DQ8 mice. These are knock- out mice which with pre-sensitization to gluten (90). Larazotide countered the intestinal barrier disintegration, decreased the macrophage count in the lamina propria, and kept the transmembrane conductance intact, in mice that were given larazotide compared to the mice who were challenged with gliadin without larazotide (91).

Phase 1 trials (NCT00362856) of Larazotide acetate were conducted on 24 CeD patients that were challenged with 2.5 g of gluten after being on a GFD for at least 6 months. The results showed that 12 mg doses of larazotide decreased the cytokine response and intestinal permeability, as seen using the urinary LAMA (lactulose to mannitol) extraction fraction, although this difference was not statistically significant. The urinary LAMA ratio is an experimental biomarker used to quantify changes in intestinal permeability in research settings. In CeD, increased permeability due to mucosal injury leads to a reduction in absorption of monosaccharides (e.g., mannitol) and an increase in the paracellular absorption of disaccharides (e.g., lactulose). This results in an increase in the ratio of lactulose to mannitol excreted in urine after oral consumption of an aqueous solution of lactulose and mannitol (92).

Phase 2a study on 86 patients assessed the effect of larazotide on urinary LAMA ratio, the gastrointestinal symptom rating scale, psychological General Well-Being Index as well as adverse event profiling. It was a double blind RCT, testing 4 doses of larazotide acetate- 0.25, 1, 4, and 8 mg in patients who had been adherent to GFD and negative to EMA and anti-tTG Ab at the baseline. There was no significant effect of larazotide on the primary efficacy endpoint after gluten challenge which was the LAMA ratio. There was a definite decrease in the gastrointestinal symptom rating scale at two doses of 0.25 and 4 mg, but not in the other dose groups of 1 and 8 mg. Larazotide was generally well-tolerated by patients with the significant adverse events being headache and urinary tract infections in more than 5% of the patients; however there were no dropouts on account of these events (93).

In Phase 2b trials, 1, 4, and 8 mg doses of enteric- coated multiparticulate beads of larazotide were given to patients in a placebo controlled study. Results showed no statistically significant difference in the LAMA levels. The 1 mg but not the 4 or 8 mg doses of drugs brought a reduction to gastrointestinal symptoms in response to gluten (94). In a later phase 2 trial which tested 0.5, 1, and 2 mg doses of larazotide (NCT01396213), primary end point of reduction of symptoms was met in the 0.25 mg dose but not in the higher doses. The pattern that has therefore emerged in all the above studies is that only the lower doses had an effect, implying an inverse-dose relationship. Peptide aggregation at higher doses of the drug, reducing activity *in vivo* may be one of the possible explanations for this observation (95). Therefore, in the near future, this drug is expected to be tested in phase 3 trials on 924 patients who have been on GFD, using enteric coated, lower doses of 0.25 mg TID and 0.5 mg TID for 16 weeks (NCT03569007).

### Approach 4: Tissue Transglutaminase- 2 Inhibition in the Intestinal Mucosa

#### Tissue Transglutaminase Inhibitors

Inhibitors of human tissue transglutaminase 2 (tTG-2) have been designed to prevent the conversion of gliadin to deamidated gliadin peptides, since the latter possess enhanced immunogenicity as antigens in CeD. tTG-2 is a multi-functional enzyme that catalyzes the linkage of glutamine and lysine side-chains to modify proteins, in the presence of ionic calcium and thiol. The enzyme is known to be associated with pathogenesis of not only CeD, but also some cancers (96) and, Parkinson's (97), Alzheimer's (98), and Huntington's diseases (99); hence tTG-2 inhibitors are being tested in many of the aforementioned conditions.

Three broad varieties of tTG-2 inhibitors have been well-described so far, namely competitive amine inhibitors, reversible inhibitors, and irreversible inhibitors. Competitive inhibitors compete with other natural amine substrates for tTG-2, thereby making the active enzyme unavailable for transamidating gliadin. These include putrescine, cystamine, spermidine, histamine, and cadaverine analogs like monodansylcadaverine. Currently the only commercially available tTG-2 inhibitor, mercaptamine or cystamine, has not been explored for its potential role in CeD (100, 101).

Reversible inhibitors inhibit substrate access to the active site without covalently modifying the tTG-2 enzyme. Examples in this class include, GTP, GDP, and their analogs, GTP- γS, βγ- methyl GTP, ionic zinc, as well as the recently discovered drugs with thieno [2,3-d]pyrimidin-4-one acyl hydrazide backbone (102).

The third group viz. irreversible inhibitors covalently bind to the cysteine in the active site and block the transglutaminase enzyme. Examples include iodoacetamide (103), -diazo-5-oxo-norleucine (DON) (104) and 3-halo-4,5-dihydroisoxazoles (105). The latter two compounds are based on the structure of acivicin, a natural analog of gliadin. Among all the above compounds, the dihydroisoxazoles and DON are selective for tTG-2 inhibition, with Ac-PQP-(DON)-LPF-NH being the most potent and selective. Also, it has been found that the 5-(S)-dihydroisoxazole is a markedly better inhibitor of human tTG-2 than its 5-(R) stereoisomer *in vitro* (106). A prototype dihydroisoxazole called 1 b, showed good oral bioavailability, efficient tTG-2 inhibition in small intestinal tissue, and low toxicity in animal studies (105). KCC009 is a dihydroisoxazole that has been studied for its application in cancers (107, 108). Other examples of dihydroisoxazole are ERW1041E (109) and R294 (110).

Also, several gluten- mimetic peptides have been developed with DON. They have a gliadin peptide sequence, where glutamines are substituted with the DON warhead (111). Gluten peptide analog ZDON (Zedira pharmaceuticals) has been shown to have a very high specificity for tTG-2. The inhibitors developed using ZDON, viz. ZED1098, ZED1219, and ZED1227 (Zedira pharmaceuticals) have demonstrated good solubility and stability in gastrointestinal conditions as well. These three compounds covalently bind to active site cysteine of tTG-2 (112). Among these, the ZED1227 has been studied in phase 1 trials. Reports suggest that it is safe and also decreases the activity of tTG-2 and inflammation of bowel mucosa in mice models (113). Recently plans for phase 2 a trial (EUDRA CT No. 2017-002241-30) were announced by Zedira pharmaceuticals.

The guiding principle in the applicability of the above inhibitors is that they should attain in the lamina propria, a sufficient concentration without inhibition of other members of the transglutaminase family. Theoretically, the inhibition of tTG-2 can result in cross inhibition of other members of the transglutaminase family, several of which are indispensable for pathways like the coagulation cascade and the maintenance of epidermal integrity. Some studies suggest that transgenic mice with tTG knockout develop autoimmune diseases like glomerulonephritis (112). Since some gluten peptides are potentially immunogenic even without deamidation by tTG, combining tTG inhibitors with other pharmaceutical agents that eliminate immunogenic peptides before they enter lamina propria would be logical.

#### Si RNA Based Therapy

Si- RNA contained in gelatin based nanoparticles have been tested *in vitro* on intestinal epithelial (Caco-2 or the continuous cell line of human colorectal adenocarcinoma) cells. These silencing RNA molecules target human tTG-2 and IL-15, both of which are incriminated in CeD pathogenesis. In a study, fluorescent microscopy revealed that these spherical particles are rapidly internalized within 2 h and localize themselves to the cytoplasm. A 60% reduction in the gene products of tTG-2 and IL-15 genes was noticed 72 h after administration, which in turn translated into reduction in IFN-γ and TNF-α levels (114). Si-RNA appears to be a promising tool, although the effects of IL-15 suppression and suppression of other glutaminases may be a cause of concern. Regardless, this approach may be emulated to block other specific targets in the CeD pathogenesis in the future as well.

### Approach 5: Prevention of Downstream Immune Activation After Gluten Exposure

#### HLA Blockers

HLA blockers prevent the interaction of antigen presenting cells (APC) that process and present gluten immunogenic epitopes using their MHC-II ligands which are a part of the HLA system, to the T Cell Receptor (TCR) of CD4+T helper cells. This amounts to prevention of an immunotoxic cascade that is a part of adaptive immunity in CeD. Cyclic peptides have been designed for blocking the gliadin binding groove of HLA-DQ2. These peptides are structurally similar to gliadin and compete with it to bind HLA-DQ2. These have the gliadin like epitopes-LQPFPQPELPY, KQPFPEKELPY, or LQLQPFPQPEKPYPQPEKPY and are cyclized using sulfide or polyethylene glycol bridges. Cyclization of these peptides results in effective blockade of the HLA grooves. Similarly, dimeric peptides with gliadin scaffolds have also demonstrated effective blockade. However, it has not translated into reduced T cell activation (115). Furthermore, ubiquitous requirement of HLA in various immune responses and a potential interference by the peptides in that function is one of the main concerns of use of HLA blockers.

#### Vaccines and Tolerogenic Therapies

The gluten products, upon entering lamina propria are recognized as immunogenic, and this triggers an adaptive immune response. Tolerogenic therapies using vaccines have been developed to hypo-sensitize the adaptive immunity.

##### Vaccine therapy

Hyposensitization is a well-known therapy for allergic diseases and is a potential therapeutic approach in autoimmune diseases as well. In a departure from their traditional use of immunization, vaccines are now being tested for desensitization. Examples of the latter include dendritic cell therapy in multiple sclerosis and in the case of Ced, the peptide-based vaccine called NexVax2. It was developed by a US based company, ImmunoSanT, Inc. NexVax2 is composed of three proprietary, immunodominant gliadin peptides named NPL001, NPL002, and NPL003 each of which is 15–16 amino acid long. The vaccine target is the HLA-DQ2.5-epitope-TCR complex linking the antigen presenting cell to the gluten-reactive CD4+ T cells.

It engages specific immune cells and a signature pathway has been discovered based on that. In animal studies in HLA-DQ2.5 transgenic mice having gluten-sensitive T cells, it was found to be efficacious (116). In a phase 1a study, 40 well-controlled CeD patients were given weekly intradermal injections of increasing doses of up to 90 μg of NexVax2 for 3 weeks. While there were some gluten-related gastrointestinal side effects, dose escalation could be completed, and safety was acceptable (117). Similarly in phase 1 b study, gradual dose escalation of up to 900 μg was tolerated well (118). Vaccinated subjects showed the target T cells becoming functionally unresponsive to antigenic stimulation with gluten challenge, consistent with immune tolerance. Nexvax2 was also associated with trends toward improved duodenal mucosal histology. Plasma concentrations of Nexvax2 peptides were dose-dependent (119).

Currently a phase 2, quadruple blind RCT (NCT03644069) is underway that will test 32 doses of twice weekly subcutaneous administration of this vaccine in subjects over a 16 week period. It will test the efficacy and improvement in CeD patient reported outcome score, as well the safety, with a follow up of 4 weeks post administration of the last dose.

The obvious limitation of vaccines is that they can engage only known or previously investigated immunogenic epitopes. Other aspects, such as efficacy and long-term safety, are to be established before peptide vaccines are made available for the management of CeD. Also, the effect profile in pediatric age group remains to be established. However, if successful, it has the potential to have prolonged benefits on patients.

##### Oral gliadin based tolerogenesis

Another way of inducing immune tolerogenesis, is the use of oral agents that act locally in the gut. *Lactobacillus lactis* has been engineered to release modified, non-toxic gliadin. A genetically engineered form of this non-colonizing, non-pathogenic bacterium was orally administered to secrete a deamidated DQ8 gliadin epitope in the intestinal lumen of transgenic NOD-2 mice with ABoDQ8 haplotype. This induced suppression of the lamina propria and systemic DQ8-restricted T-cell responses, downregulation of IL-12 secretion, systemic production of IL-10 and TGF-β and induction of Foxp3+ Tregs in the lamina propria. These findings suggest development of mucosal tolerance to the gliadin (120).

Similarly a study used *Bacillus subtilis* spores as a long-lived, protease-resistant adjuvant system for administering gliadin peptides to HLA-DQ8-transgenic mice. The spore-adsorbed gliadin activated the dendritic cells and elicited a T-cell response in the gut. This mechanism (121) can be utilized for developing immune tolerance.

##### Tolerogenic immunomodulatory peptides.

Tolerogenic immunomodulatory peptides (TIMPs) are nano particles that may be used to deliver peptide epitopes intravenously to induce systemic immune tolerance. Gliadin containing TIMPs has shown favorable results in a single animal study (122). A drug called TIMP-GLIA (Cours pharmaceuticals) has been developed along these lines, having acquired a fast track approval from the FDA for phase 1 testing. The results of phase 1 trial are yet to be published, and the phase 2 trial is currently underway.

#### Cathepsin Inhibitor

Cathepsin is an intracellular protease that mediates apoptosis. A type of cathepsin called cathepsin S is expressed specifically in antigen presenting cells (APCs) where it mediates the proteolysis of the invariant chain. Invariant chain is an intracellular protein that prevents intracytoplasmic, self- antigen loading during the early stages of development of the MHC-II molecules of APCs. Once the MHC-II complex matures intracellularly, cathepsin S cleaves the invariant chain to permit antigen loading. Hence cathepsin S is important for the MHC II to function normally to present processed antigens to CD4+ cells (123). This mechanism is central to autoimmunity.

The cathepsin inhibitor, RO5459072 (also called RG7625), has been developed to target this pathway and is being tested in CeD, Sjogren's syndrome and other autoimmune diseases. A study (NCT022953320) showed that this drug resulted in a decrease in maturation of MHC-II bearing B cells and dendritic cells (124). The effects of RO5459072 on the immune response to gluten challenge in CeD patients has been investigated in a phase 1, placebo-controlled RCT (NCT02679014). Volunteers with previously diagnosed CeD were randomized to receive either 100 mg of RO5459072 or placebo twice daily for 28 days. The results of the study are awaited. Another cathepsin inhibitor in the pipeline is RG7236.

#### Immunosuppressants

CeD is similar to inflammatory bowel disease in being a chronic inflammatory condition with a similar profile of inflammatory chemical mediators. A major difference is that in CeD, abrogation of immune cascade occurs with GFD, IBD on the other hand requires lifelong immunosuppression. However, a subset of CeD patients require immunosuppression as an adjunct to GFD to enhance their recovery; these are patients suffering from refractory CeD, celiac crisis, and gliadin shock. The following are some of the anti-inflammatory and immune modulating drugs are being investigated in the context of CeD.

##### Glucocorticoids

Early historic reports on the inhibitory effect of steroids on B and T cell proliferation and on release of lymphokines by cultured cells based on *in vitro* studies, indicated that steroids may be effective in CeD (125). Wall et al. then showed an accelerated improvement in symptoms by addition of prednisone to GFD in patients with CeD (126). In another *in vitro* study, Mitchison et al. had also demonstrated the preventive effects of fluticasone propionate on the immunotoxicity of gluten (127).

Among the later studies, a proof of concept RCT evaluated the effects of a short course of prednisolone along with GFD on the markers of apoptosis and anti-apoptosis pathways, as well as on epithelial cell regeneration in treatment naïve CeD patients. The hypothesis was that addition of prednisolone for a short term, would accentuate the mucosal recovery. A 4 week course of 1 mg/kg/d prednisolone in addition to GFD was given to treatment naïve CeD patients. It was found that compared to baseline, patients on both GFD alone control group as well as the GFD plus prednisolone group had underexpression of the markers of apoptosis viz. p53 and M30. However, all apoptotic markers other than H2AX showed a rebound increase 4 weeks after cessation of steroids, indicating that steroids may have indeed played a role in suppressing apoptosis and epithelial injury, during the period where the steroid was available in the systemic circulation. The study also found that steroids could however counterproductively lead to a suppression of proliferation index and therefore of epithelial regeneration (128).

More recently, budesonide has piqued the interest of researchers in view of its potential application as a steroid with low systemic bioavailability and hence higher effect on the gut. Ciacci et al. studied the effect of oral administration of 6 mg budesonide capsules for 4 weeks on CeD patients on GFD. It was found that the drug significantly decreased stool frequency and stool weight and improved general well-being as indicated by subjects on a Visual Analog Scale, in comparison to those on GFD alone. The same study also investigated the effect of budesonide *in vitro* on intestinal epithelial cells from biopsies of CeD patients, exposed to gliadin and its toxic fraction p21-43. Budesonide significantly decreased the expression of inflammatory markers ICAM-1, COX-2, and HLA-DR (129).

##### Steroids in refractory CeD

Brar et al. studied retrospectively, 30 CeD patients who were prescribed budesonide while following up for symptoms that were refractory despite maintaining adherence to GFD. They noticed, that 75% of patients had at least partial response to budesonide ± either azathioprine or systemic steroids, and 55% responded completely. Complete response was higher among those with secondary refractory CeD i.e., in those whose disease manifestations recurred after an initial response to a GFD, compared to those whose disease remained refractory from the time of institution of the GFD (primary refractory CeD) (130).

In another recent series, Mukewar et al. showed clinical (92%) and histologic (89%) improvement in 57 patients with refractory CeD who were treated with oral budesonide. Half of these patients had earlier been treated with immunosuppressive therapy with no or incomplete response. Follow-up biopsy in 7 out of 13 patients with refractory CeD-2 (RCD-2) (53%) showed an absence of clonal TCR gamma gene rearrangement/aberrant IEL phenotype that was previously seen (131). Similar response was also reported by Daum in patients with refractory CeD (132).

##### Stem cell therapy

Intestinal epithelial cells in the crypts and villi are in a state of continuous turnover with stem cells from the base of crypts proliferating, differentiating into mature phenotypes and rising to the outer villous surface to replenish the older cells that are shed. These stem cells are identified by the markers CD133+/Lgr5+. There have been case reports of using hematopoietic autologous stem cell transplant being successfully used to treat the enteropathy of CeD patients (133). Autologous stem cell transplant has been tried in patients with refractory CeD as well. In a pilot study, 13 patients with refractory CeD were subjected to ASCT, it was found that not only was ASCT well tolerated, but also it led to rapid clinical response which lasted for at least 2 years in patients (134). Furthermore, in a series amongst 54 patients who had been diagnosed with enteropathy associated T-cell lymphoma from 1994 to 1998, 14 underwent ASCT. All of them showed disappearance of intestinal lesions as well as prolonged remission of the disease (135). However, occurrence of neutropenia and other complications of stem cell transplant are likely to affect the acceptability of this therapy except in some refractory cases.

##### CCR9 antagonists

In order for immunoreactive T cells to home in to the intestinal mucosa and cause celiac autoimmunity, they use their ligand called CCR9 to bind with the receptor CCL25 on the intestinal mucosal epithelium. Antagonists to CCR9 have been developed, e.g., CCX8037 and GSK-1605786 (CCX-282; Traficet-EN, Vercirnon). The latter is a drug that is being developed for potential use in Crohn's disease and CeD. This molecule was characterized and tested *in vitro* by Walters et al. on Molt-4-T cell lines which endogenously express CCR9. Stimulation of Molt-4 cells with CCL25 resulted in release of intracellular ionic calcium which in turn promoted chemotaxis. Also, CCL25 is a chemoreceptor specific to intestinal mucosa. The antagonist to CCL25 prevented the interaction of chemokines CCL25 and CCR9, and prevented release of calcium and homing- in of inflammatory T cells. The same study also found the results to be replicable in mouse models where intestinal inflammation was attenuated in response to the drug. Furthermore, the drug CCX282 was found to be highly selective for CCL25-CCR9 interaction (136). Tubo et al. have also tested this drug in mouse models and found similar results. Interestingly they also inoculated this drug into inflamed ear skin in mice, and found no attenuation of inflammatory response in the draining lymph nodes showing that this drug would specifically act in the immune system of the intestine and spare other immune organs (137).

A RCT (NCT00540657) to study the efficacy of administration of 250 mg of oral CCX282 twice daily in comparison to placebo has been completed in patients with CeD on 24 months of GFD. The outcome measures included effects on V:C ratio and markers of intestinal inflammation, serology and symptoms upon gluten exposure. The results of this study are yet to be published. While a phase 2 clinical trial of CCX282 in patients with Crohn's disease (NCT00306215) had given encouraging results (138), the phase 3 trials failed to show any significant difference of results compared to placebo (139).

##### Interleukin-15 antagonists.

IL-15 is overexpressed in the intestinal mucosa in CeD on exposure to gluten. It mediates the inflammatory response that leads to intestinal epithelial damage. Humanized Mik-Beta-1 monoclonal antibody has been developed to target IL-2/IL-15R Beta (CD122). Hu-Mik- Beta-1 is currently being investigated for safety in phase 1 trials in a three dose regimen (Day 1, week 3, week 6) in refractory CeD patients.

Another human monoclonal antibody against IL-15, with a similar mechanism of action is AMG 714 (140). This drug has been developed by the biotechnology company, Amgen. It is being investigated in phase 2, double blind placebo controlled, dose- varying RCT.

##### Integrin antagonists.

Vedolizumab is a humanized monoclonal antibody that prevents the interaction of anti-α4β7 integrin with mucosal addressin cell adhesion molecule-1 (MAdCAM-1). The latter is found exclusively on the intestinal mucosa, enabling vedolizumab to prevent chemotaxis of memory T cells into the mucosa from the circulation. This specific chemotaxis directed toward mucosa or homing- in of T cells is responsible for the chronic inflammation seen in IBD. Vedolizumab is therefore used for the management of patients with Crohn's disease and ulcerative colitis. Similar to IBD, there is an overexpression of α4β7 integrin in patients with CeD and hence its effect in CeD is worth exploring. Vedolizumab is under a phase 2 clinical trial (NCT02929316) in CeD patients, with results expected in 2019.

##### Immunomodulator drugs with potential application in CeD

Other potential immunomodulators include anti- TNF-α agents like infliximab. Infliximab has been shown to be useful in case reports of refractory CeD (141, 142), however a larger human trial is yet to be done. Similarly natalizumab has also been tried in patients with multiple sclerosis or IBD in addition to CeD (143), with induction of remission in both CeD and the co-occurring condition.

Mulder et al. studied the use of recombinant IL-10 for treating patients with refractory CeD and found that only 2 of the 10 treated patients responded histologically (144). Another potential immunomodulator is the anti-CD20 antibody, rituximab. While it is worth exploring the use of this agent in CeD to abrogate the abnormal mucosal plasmablasts and antibody formation, a study found no significant effect of rituximab on mucosal B cells (145).

### Other Therapies Under Investigation

Besides the five approaches discussed in the preceding sections, there are a few experimental therapies that modulate factors known to be associated with CeD but as yet are not known to play an obvious role in its pathogenesis. These therapies include probiotics and prebiotics, the low FODMAP diet, pancreatic enzyme supplements, and helminth therapy using hookworms. Probiotics have been tried in patients with CeD, since studies have found a reduction in the relative proportion of Firmicutes, Proteobacteria, Bifidobacterium and a relative increase in Bacteroides and E.Coli in CeD patients compared to controls. A few RCTs have found that probiotics lead to improvement in symptoms in CeD (146) while some animal studies have found *Bifidobacterium* and *Lactobacillus* to reduce gluten-induced toxicity. The proprietary probiotic formulation VSL#3 has demonstrated activity in gliadin pre-digestion in a study (147). Similarly, a study found that oligofructose-enriched inulin (Synergy 1), a prebiotic, increased the Bifidobacterium count in the gut significantly, with no side effects (148). These findings point to a possible causative role of gut dysbiosis in CeD, although the exact mechanism remains obscure. Studies are also exploring the use of low FODMAP (Fermentable oligo-, di-, mono- saccharides, and polyols) diet, which is low in short chain polysaccharides like fructans, lactose, mannitol, sorbitol etc. These sugars are hard to digest, resulting in fermentation in the bowel and flatulence, and are implicated in causing some of the symptoms of Irritable Bowel Syndrome (IBS) (149). The low FODMAP diet may therefore be beneficial in CeD, especially in those with functional IBS like (150–152) symptoms. One limitation of this diet is that it is even more restrictive than GFD and can increase susceptibility to nutritional deficiencies. It is also noteworthy that much of the evidence concerning the use of low FODMAP diet is derived from moderate to low quality studies in IBS patients where proper blinding and controls have not been used, with uncertainty over whether the benefit of the diet is a placebo effect (153). In another experimental approach, Weiss tested the hygiene hypothesis of autoimmunity by investigating the effect of hookworm *(Necator americanus)* therapy on CeD. The hygiene hypothesis has been derived from the observation that there has been a simultaneous decrease in infectious diseases and an increase in autoimmune diseases globally (154). Chronic low grade helminthiasis, while inducing specific immune response to itself, also diminishes Th1 cells response to other antigens (155), which can in turn suppress autoimmunity. Daveson found that inoculation of hookworms into the skin increased microbial richness in the fecal samples in the same patients. It is possible therefore that helminthic infection modulates CeD pathogenesis through an unknown mechanism that could be similar to probiotics (156–159). Furthermore, they found hookworm therapy to be safe and well-tolerated with none of the patients developing significant anemia. Administration of pancreatic enzyme supplements may also be helpful in a subset of CeD patients classified as having Non Responsive Celiac Disease or NRCD (persisting symptoms of CeD despite 6–12 months of adhering to a GFD). This is because exocrine pancreatic insufficiency may be one of the factors known to contribute to NRCD symptoms, other factors being microscopic colitis, refractory CeD, dietary lactose/fructose malabsorption and small intestinal bacterial overgrowth (147). A RCT is currently underway to test the effect of pancreatic enzyme supplementation in NRCD patients (NCT02475369).

## Conclusions

GFD is likely to remain the mainstay of therapy of CeD in the near future, since all other treatment modalities are only in the preliminary stages of research. An ideal therapeutic agent would be one that permits a CeD patient to consume gluten in usual amounts, without compromising her/his quality of life. So far, only vaccines have come closest to the potential of helping patients achieve that ideal state. Vaccines also show promise in terms of bearing prolonged benefits, subverting the need for gluten restriction all together. Glutenases are another group of drugs that have been extensively explored as therapeutic agents and their list is growing with newer discoveries. Among these, latiglutenase had reached the farthest in terms of clinical trials; although the most recent clinical trial on a large sample size delivered disappointing results. Results of preliminary studies on another glutenase, Kuma030, suggest that this enzyme may hold more promise in future, compared to other glutenases studied so far. Similarly, large scale testing of the zonulin antagonist, larazotide acetate with lower doses could yield new insights into its effectiveness, due to results of previous smaller studies demonstrating an inverse dose- response relationship. To conclude, although most trials on novel therapeutics are currently in phase 2 or earlier stages, ongoing research in areas targeting various molecular pathways in CeD is robust. This leaves much scope to find definitive alternatives to GFD in the years to come, in order to improve the quality of life of patients with CeD.

## Author Contributions

SY: review of the literature, drafting of the manuscript, conceptualization of illustrations. GM: concept building, critical review and finalization of the manuscript.

### Conflict of Interest Statement

The authors declare that the research was conducted in the absence of any commercial or financial relationships that could be construed as a potential conflict of interest.
