# Supplementary material for: Evolving Therapy for Celiac Disease
Source: Front Pediatr. 2019 May 14;7:193. doi: 10.3389/fped.2019.00193 (PMC6530343; doi:10.3389/fped.2019.00193)
Supplement: Supplementary file 1 [file Table_1.docx]

**Supplementary Table. Relevant studies and trials in the area of emerging therapeutics for celiac disease**

| Mechanism of action/ Drug class | Drug | Trial phase | Trial identifier number | Sponsor/ authors | Study period */ time of publication | Reference *** |
| --- | --- | --- | --- | --- | --- | --- |
| Glutenase | AN-PEP | 1 | NCT00810654 | VU University Medical Center | May 2008- December 2009 | (1) |
|  |  | Not applicable | NCT01335503 | DSM Food Specialties | April 2011- May 2012 | (2) |
|  |  | *In vitro* |  | Stepniak et al. | 2006 | (3) |
|  | STAN 1 | 1 | NCT00962182 | Heim Pal Children's Hospital | December 2014-December 2017 | ** |
|  | ALV 003 | 1 | NCT00669825 | Alvine pharmaceuticals | March 2008- October 2008 | ** |
|  |  | 1 | NCT00626184 |  | February 2008- July 2008 | ** |
|  |  | 2a | NCT00959114 |  | August 2009- October 2010 | (4) |
|  |  | 2a | NCT01255696 |  | November 2010- June 2011 | (4) |
|  |  | 2b | NCT01917630 |  | August 2013- June 2015 | (5),(6) |
|  |  | 2b | NCT03585478 |  | July 2018- May 2020* |  |
|  |  | *In vitro* | NCT00859391 |  | May 2008-June 2008 | (7) |
| Modified gluten | Gluten friendly (GLUFR) bread | *In vivo* | NCT03168490 | University of Roehampton, London, UK | February 2016- April 2018 | ** |
|  |  | 2 | NCT03137862 | University of Foggia, Foggia, Italy | March 2017- January 2018 | ** |
|  | Enzyme digested gluten bread | 2 | NCT02472119 | University of Roma La Sapienza, Rome, Italy | June 2015- August 2015 | (8) |
| Paracellular gliadin transport antagonist (Zonulin antagonist) | Larazotide acetate (AT-1001) | 3 | NCT03569007 | Innovate Biopharmaceuticals | October2018*- December 2020* |  |
|  |  | 2b | NCT01396213 |  | November 2007- August 2013 | (9) |
|  |  | 2b | NCT00889473 |  | April 2009- April 2010 | ** |
|  |  | 2b | NCT00620451 |  | February 2008- December 2009 | ** |
|  |  | 2b | NCT00492960 |  | August 2007- March 2009 | (10) |
|  |  | 1/2a | NCT00362856 |  | September 2006- March 2007 | (11),(12) |
|  |  | 1 | NCT00386490 |  | January 2006- February 2006 | ** |
|  |  | 1b | NCT00386165 |  | November 2005-January 2006 | ** |
| Transcellular gliadin transport anatagonist  (Anti CD71- sIgA pathway) |  | *In vitro*, *in vivo* |  | Lebreton et al. | 2010 | (13) |
| Immunotolerance to gliadin | NexVax-2 (vaccine) | 2 | NCT03644069 | ImmusanT, Inc. | August 2018- September 2019* |  |
|  |  | 1 | NCT03543540 |  | May 2018- October 2018* |  |
|  |  | 1 | NCT02528799 |  | August 2015-January 2017 | (14) |
|  |  | 1 | NCT00879749 | Nexpep Pty Ltd | April 2009-March 2010 | (15) |
|  | Oral *Lactobacillus lactis* secreting non-toxic gliadin | *In vivo* |  | Huibregtse et al. | 2009 | (16) |
|  | Oral Bifidobacterium adsorbed to gliadin | *In vivo* |  | Laparra et al. | 2013 | (17) |
|  |  | *In vitro* |  |  | 2010 | (18) |
|  | TIMP-GLIA (Tolerogenic Immuno- Modulatory Peptides) | 1 | NCT03486990 | Cours pharma. | February 2018- March 2019* |  |
| Gliadin sequestration | BL-7010 or p(HEMA- co-SS) | 1 | NCT01990885 | BioLineRx, Ltd. | December 2013- October 2014 | ** |
|  |  | Animal study |  | McCarville et al. | 2014 | (19) |
|  |  | Animal study |  | Pinier et al. | 2012 | (20) |
|  |  | *In vitro* |  |  | 2009 | (21) |
| Recombinant tandem single chain fragment variable |  | Not applicable |  | Eggenreich | November 2016- March 2018 | (22) |
| Anti-gluten antibody | Oral AGY | 1 | NCT01765647 | Igy Inc. | May 2014- August 2015 | (23) |
| Transglutaminase inhibitors | KCC009 | Discovery |  | Choi et al. | 2005 | (24) |
|  | ZED 101 | *In vitro* |  | Zedira pharma. |  |  |
| SiRNA- based gene silencing | Gelatin nanoparticles to deliver siRNA acting on tTG and IL-15 | *In vitro* |  | Attarwala et al. | 2017 | (25) |
| Cathepsin inhibitor | RO5459072 (RG7625) | 1 | NCT02679014 | Hoffmann-La Roche | March 2016- August 2016 | ** |
| HLA-DQ2 blockers | Cyclic and dimeric gluten peptide analogs | *In vitro* |  | Xia et al. | 2007 | (26) |
|  | Peptide | Discovery |  | Kapoerchan et al. | 2010 | (27) |
|  | Azidoproline containing gluten peptides | Discovery |  |  | 2008 | (28) |
| Steroids | Prednisolone | 2 | [NCT01045837](http://clinicaltrials.gov/show/NCT01045837) | All India Institute of Medical Sciences, New Delhi, India | April 2009- August 2010 | (29) |
|  |  | 2 | CTRI/2017/08/009517 | Jawaharlal Nehru Medical College, Aligarh, India | March 2010- October 2012 | (30) |
|  | Budesonide | *In vitro*/ Phase 2 |  | Ciacci et al. | 2009 | (31) |
| Hookworm therapy | *Necator americanus* | 2 | NCT00671138 | James Cook University, Queensland, Australia | August 2012- March 2014 | (32,33) |
|  |  | 1b | [NCT02754609](https://clinicaltrials.gov/show/NCT02754609) |  | September 2016- December 2020* |  |
|  |  | 1b/2a | NCT01661933 |  | October 2007- September 2009 | ** |
| CCR9 antagonist | CCX282B | 2 | NCT00540657 | ChemoCentryx | October 2007- July 2008 | ** |
|  | CCR9 antagonist | Preclinical |  | Walters | 2010 | (34,35) |
| Anti-IL-15 antibody | Hu-Mik- Beta-1 | 1 | NCT01893775 | National Cancer Institute (NCI) | June 2013- June 2021* |  |
|  | AMG 714 | 2a | NCT02637141 | Amgen | March 2016- March 2017 | ** |
|  |  |  | NCT00433875 |  | December 2002-December 2007 | (36) |
| Anti-integrin antibody | Vedolizumab | 2 | NCT02929316 | AGA Clinical Research Associates, LLC | June 2018-December 2018* |  |
| Low FODMAP (Fermentable Oligo-, Di-, Mono- saccharides and Polyols) diet |  | Not applicable | [NCT03644602](https://clinicaltrials.gov/show/NCT03644602) | Federico II University, Naples, Italy | July 2016-December 2016 | (37) |
|  |  | Not applicable | NCT02946827 | Fondazione IRCCS Ca' Granda, Ospedale Maggiore Policlinico, Italy | January 2016- January 2017 | ** |
| Prebiotic/ probiotic | Synergy1 (oligo-fructose enriched inulin) | Not applicable | [NCT03064997](https://clinicaltrials.gov/show/NCT03064997) | Polish Academy of Sciences, Warsaw, Poland | January 2016- June 2016 | (38,39) |
|  | Protalsafe | Not applicable | NCT03168490 | Société Guaranteed Gluten Free (GGF), France | December 2016- January 2018 | ** |
|  | NLS super strain (Natren LIFE START®2) | 2 | NCT03271138 | Global Institute of Probiotics, Argentina | July 2017-December 2017 | ** |
|  | *Bifidobacterium infantis* | Not applicable | NCT01257620 | Julio Bai M.D., Argentina | December 2010- December 2011 | (40) |
|  | *Triticum monococcum* | 2 | NCT02220166 | Università degli Studi di Brescia | November 2010- August 2011 | ** |
|  | ProCel (Probiotic mixture) | Not applicable | NCT01699191 | University of Bari | 2013-2015 | (41) |
| Pancreatic enzyme supplementation | Pancrelipase | 4 | NCT02475369 | Beth Israel Deaconess Medical Center | May 2015- December 2019* |  |

*Estimated time for study period

** As of August 2018, results were not available despite completion of the study

*** References:

1. Tack GJ, van de Water JMW, Bruins MJ, Kooy-Winkelaar EMC, van Bergen J, Bonnet P, et al. Consumption of gluten with gluten-degrading enzyme by celiac patients: a pilot-study. World J Gastroenterol. 2013 Sep 21;19(35):5837–47.

2. Salden BN, Monserrat V, Troost FJ, Bruins MJ, Edens L, Bartholomé R, et al. Randomised clinical study: Aspergillus niger-derived enzyme digests gluten in the stomach of healthy volunteers. Aliment Pharmacol Ther. 2015 Aug;42(3):273–85.

3. Stepniak D, Spaenij-Dekking L, Mitea C, Moester M, de Ru A, Baak-Pablo R, et al. Highly efficient gluten degradation with a newly identified prolyl endoprotease: implications for celiac disease. Am J Physiol Gastrointest Liver Physiol. 2006 Oct;291(4):G621-629.

4. Lähdeaho M-L, Kaukinen K, Laurila K, Vuotikka P, Koivurova O-P, Kärjä-Lahdensuu T, et al. Glutenase ALV003 attenuates gluten-induced mucosal injury in patients with celiac disease. Gastroenterology. 2014 Jun;146(7):1649–58.

5. Murray JA, Kelly CP, Green PHR, Marcantonio A, Wu T-T, Mäki M, et al. No Difference Between Latiglutenase and Placebo in Reducing Villous Atrophy or Improving Symptoms in Patients With Symptomatic Celiac Disease. Gastroenterology. 2017;152(4):787-798.e2.

6. Syage JA, Murray JA, Green PHR, Khosla C. Latiglutenase Improves Symptoms in Seropositive Celiac Disease Patients While on a Gluten-Free Diet. Dig Dis Sci. 2017;62(9):2428–32.

7. Tye-Din JA, Anderson RP, Ffrench RA, Brown GJ, Hodsman P, Siegel M, et al. The effects of ALV003 pre-digestion of gluten on immune response and symptoms in celiac disease in vivo. Clin Immunol. 2010 Mar;134(3):289–95.

8. Marino M, Casale R, Borghini R, Di Nardi S, Donato G, Angeloni A, et al. The effects of modified versus unmodified wheat gluten administration in patients with celiac disease. Int Immunopharmacol. 2017 Jun;47:1–8.

9. Leffler DA, Kelly CP, Green PHR, Fedorak RN, DiMarino A, Perrow W, et al. Larazotide acetate for persistent symptoms of celiac disease despite a gluten-free diet: a randomized controlled trial. Gastroenterology. 2015 Jun;148(7):1311-1319.e6.

10. Kelly CP, Green PHR, Murray JA, Dimarino A, Colatrella A, Leffler DA, et al. Larazotide acetate in patients with coeliac disease undergoing a gluten challenge: a randomised placebo-controlled study. Aliment Pharmacol Ther. 2013 Jan;37(2):252–62.

11. Leffler DA, Kelly CP, Abdallah HZ, Colatrella AM, Harris LA, Leon F, et al. A Randomized, Double-Blind Study of Larazotide Acetate to Prevent the Activation of Celiac Disease During Gluten Challenge. Am J Gastroenterol. 2012 Oct;107(10):1554–62.

12. Paterson BM, Lammers KM, Arrieta MC, Fasano A, Meddings JB. The safety, tolerance, pharmacokinetic and pharmacodynamic effects of single doses of AT-1001 in coeliac disease subjects: a proof of concept study. Aliment Pharmacol Ther. 2007 Sep 1;26(5):757–66.

13. Lebreton C, Ménard S, Abed J, Moura IC, Coppo R, Dugave C, et al. Interactions among secretory immunoglobulin A, CD71, and transglutaminase-2 affect permeability of intestinal epithelial cells to gliadin peptides. Gastroenterology. 2012 Sep;143(3):698-707.e4.

14. Daveson AJM, Ee HC, Andrews JM, King T, Goldstein KE, Dzuris JL, et al. Epitope-Specific Immunotherapy Targeting CD4-Positive T Cells in Celiac Disease: Safety, Pharmacokinetics, and Effects on Intestinal Histology and Plasma Cytokines with Escalating Dose Regimens of Nexvax2 in a Randomized, Double-Blind, Placebo-Controlled Phase 1 Study. EBioMedicine. 2017 Dec;26:78–90.

15. Tye-Din JA, Stewart JA, Dromey JA, Beissbarth T, van Heel DA, Tatham A, et al. Comprehensive, quantitative mapping of T cell epitopes in gluten in celiac disease. Sci Transl Med. 2010 Jul 21;2(41):41ra51.

16. Huibregtse IL, Marietta EV, Rashtak S, Koning F, Rottiers P, David CS, et al. Induction of antigen-specific tolerance by oral administration of Lactococcus lactis delivered immunodominant DQ8-restricted gliadin peptide in sensitized nonobese diabetic Abo Dq8 transgenic mice. J Immunol. 2009 Aug 15;183(4):2390–6.

17. Laparra JM, Olivares M, Sanz Y. Oral administration of Bifidobacterium longum CECT 7347 ameliorates gliadin-induced alterations in liver iron mobilisation. Br J Nutr. 2013 Nov;110(10):1828–36.

18. Laparra JM, Sanz Y. Bifidobacteria inhibit the inflammatory response induced by gliadins in intestinal epithelial cells via modifications of toxic peptide generation during digestion. J Cell Biochem. 2010 Mar 1;109(4):801–7.

19. McCarville JL, Nisemblat Y, Galipeau HJ, Jury J, Tabakman R, Cohen A, et al. BL-7010 demonstrates specific binding to gliadin and reduces gluten-associated pathology in a chronic mouse model of gliadin sensitivity. PLoS ONE. 2014;9(11):e109972.

20. Pinier M, Fuhrmann G, Galipeau HJ, Rivard N, Murray JA, David CS, et al. The copolymer P(HEMA-co-SS) binds gluten and reduces immune response in gluten-sensitized mice and human tissues. Gastroenterology. 2012 Feb;142(2):316-325.e1-12.

21. Pinier M, Verdu EF, Nasser-Eddine M, David CS, Vézina A, Rivard N, et al. Polymeric binders suppress gliadin-induced toxicity in the intestinal epithelium. Gastroenterology. 2009 Jan;136(1):288–98.

22. Eggenreich B, Scholz E, Wurm DJ, Forster F, Spadiut O. The production of a recombinant tandem single chain fragment variable capable of binding prolamins triggering celiac disease. BMC Biotechnol. 2018 May 29;18(1):30.

23. Sample DA, Sunwoo HH, Huynh HQ, Rylance HL, Robert CL, Xu B-W, et al. AGY, a Novel Egg Yolk-Derived Anti-gliadin Antibody, Is Safe for Patients with Celiac Disease. Dig Dis Sci. 2017;62(5):1277–85.

24. Choi K, Siegel M, Piper JL, Yuan L, Cho E, Strnad P, et al. Chemistry and biology of dihydroisoxazole derivatives: selective inhibitors of human transglutaminase 2. Chem Biol. 2005 Apr;12(4):469–75.

25. Attarwala H, Clausen V, Chaturvedi P, Amiji MM. Cosilencing Intestinal Transglutaminase-2 and Interleukin-15 Using Gelatin-Based Nanoparticles in an in Vitro Model of Celiac Disease. Mol Pharm. 2017 Sep 5;14(9):3036–44.

26. Xia J, Bergseng E, Fleckenstein B, Siegel M, Kim C-Y, Khosla C, et al. Cyclic and dimeric gluten peptide analogues inhibiting DQ2-mediated antigen presentation in celiac disease. Bioorg Med Chem. 2007 Oct 15;15(20):6565–73.

27. Kapoerchan VV, Wiesner M, Hillaert U, Drijfhout JW, Overhand M, Alard P, et al. Design, synthesis and evaluation of high-affinity binders for the celiac disease associated HLA-DQ2 molecule. Mol Immunol. 2010 Feb;47(5):1091–7.

28. Kapoerchan VV, Wiesner M, Overhand M, van der Marel GA, Koning F, Overkleeft HS. Design of azidoproline containing gluten peptides to suppress CD4+ T-cell responses associated with celiac disease. Bioorg Med Chem. 2008 Feb 15;16(4):2053–62.

29. Shalimar null, Das P, Sreenivas V, Datta Gupta S, Panda SK, Makharia GK. Effect of addition of short course of prednisolone to gluten-free diet on mucosal epithelial cell regeneration and apoptosis in celiac disease: a pilot randomized controlled trial. Dig Dis Sci. 2012 Dec;57(12):3116–25.

30. Abbas A, Shahab T, Sherwani RK, Alam S. Addition of a Short Course of Prednisolone to a Gluten-Free Diet vs. Gluten-Free Diet Alone in Recovery of Celiac Disease: A Pilot Randomized Controlled Trial. Cureus. 2018 Jan 28;10(1):e2118.

31. Ciacci C, Maiuri L, Russo I, Tortora R, Bucci C, Cappello C, et al. Efficacy of budesonide therapy in the early phase of treatment of adult coeliac disease patients with malabsorption: an in vivo/in vitro pilot study. Clin Exp Pharmacol Physiol. 2009 Dec;36(12):1170–6.

32. Daveson AJ, Jones DM, Gaze S, McSorley H, Clouston A, Pascoe A, et al. Effect of hookworm infection on wheat challenge in celiac disease--a randomised double-blinded placebo controlled trial. PLoS ONE. 2011 Mar 8;6(3):e17366.

33. Cantacessi C, Giacomin P, Croese J, Zakrzewski M, Sotillo J, McCann L, et al. Impact of experimental hookworm infection on the human gut microbiota. J Infect Dis. 2014 Nov 1;210(9):1431–4.

34. Walters MJ, Wang Y, Lai N, Baumgart T, Zhao BN, Dairaghi DJ, et al. Characterization of CCX282-B, an orally bioavailable antagonist of the CCR9 chemokine receptor, for treatment of inflammatory bowel disease. J Pharmacol Exp Ther. 2010 Oct;335(1):61–9.

35. Ciccocioppo R, Gallia A, Avanzini MA, Betti E, Picone C, Vanoli A, et al. A Refractory Celiac Patient Successfully Treated With Mesenchymal Stem Cell Infusions. Mayo Clin Proc. 2016 Jun;91(6):812–9.

36. Lebrec H, Horner MJ, Gorski KS, Tsuji W, Xia D, Pan W-J, et al. Homeostasis of human NK cells is not IL-15 dependent. J Immunol. 2013 Dec 1;191(11):5551–8.

37. Testa A, Imperatore N, Rispo A, Rea M, Tortora R, Nardone OM, et al. Beyond Irritable Bowel Syndrome: The Efficacy of the Low Fodmap Diet for Improving Symptoms in Inflammatory Bowel Diseases and Celiac Disease. Dig Dis. 2018;36(4):271–80.

38. Krupa-Kozak U, Drabińska N, Jarocka-Cyrta E. The effect of oligofructose-enriched inulin supplementation on gut microbiota, nutritional status and gastrointestinal symptoms in paediatric coeliac disease patients on a gluten-free diet: study protocol for a pilot randomized controlled trial. Nutr J. 2017 Aug 22;16(1):47.

39. Drabińska N, Jarocka-Cyrta E, Markiewicz LH, Krupa-Kozak U. The Effect of Oligofructose-Enriched Inulin on Faecal Bacterial Counts and Microbiota-Associated Characteristics in Celiac Disease Children Following a Gluten-Free Diet: Results of a Randomized, Placebo-Controlled Trial. Nutrients [Internet]. 2018 Feb 12 [cited 2018 Sep 23];10(2). Available from: https://www.ncbi.nlm.nih.gov/pmc/articles/PMC5852777/

40. Smecuol E, Hwang HJ, Sugai E, Corso L, Cherñavsky AC, Bellavite FP, et al. Exploratory, randomized, double-blind, placebo-controlled study on the effects of Bifidobacterium infantis natren life start strain super strain in active celiac disease. J Clin Gastroenterol. 2013 Feb;47(2):139–47.

41. Francavilla R, Piccolo M, Francavilla A, Polimeno L, Semeraro F, Cristofori F, et al. Clinical and Microbiological Effect of a Multispecies Probiotic Supplementation in Celiac Patients With Persistent IBS-type Symptoms: A Randomized, Double-Blind, Placebo-controlled, Multicenter Trial. J Clin Gastroenterol. 2018 Apr 23;

-------------------------------------------------------------------------------------------------------------------------------
